# Synthesis, characterisation, biological evaluation and *in silico* studies of sulphonamide Schiff bases

**DOI:** 10.1080/14756366.2020.1746784

**Published:** 2020-04-05

**Authors:** Mustafa Durgun, Cüneyt Türkeş, Mesut Işık, Yeliz Demir, Ali Saklı, Ali Kuru, Abdussamat Güzel, Şükrü Beydemir, Suleyman Akocak, Sameh M. Osman, Zeid AlOthman, Claudiu T. Supuran

**Affiliations:** a Department of Chemistry, Faculty of Arts and Sciences, Harran University, Şanlıurfa, Turkey; b Department of Biochemistry, Faculty of Pharmacy, Erzincan Binali Yıldırım University, Erzincan, Turkey; c Department of Pharmacy Services, Vocational School of Health Services, Harran University, Şanlıurfa, Turkey; d Department of Pharmacy Services, Nihat Delibalta Göle Vocational High School, Ardahan University, Ardahan, Turkey; e Department of Chemistry, Faculty of Arts and Sciences, Sakarya University, Sakarya, Turkey; f Department of Pharmacy Services, Vocational School of Health Services, İnönü University, Malatya, Turkey; g Department of Biochemistry, Faculty of Pharmacy, Anadolu University, Eskişehir, Turkey; h Department of Pharmaceutical Chemistry, Faculty of Pharmacy, Adiyaman University, Adiyaman, Turkey; i Department of Chemistry, King Saud University, Riyadh, Kingdom of Saudi Arabia; j NEUROFARBA Department, Sezione di Scienze Farmaceutiche, Universita degli Studi di Firenze, Florence, Italy

**Keywords:** Acetylcholinesterase, carbonic anhydrase, synthesis, sulphonamide, molecular docking

## Abstract

Sulphonamides are biologically important compounds with low toxicity, many bioactivities and cost-effectiveness. Eight sulphonamide derivatives were synthesised and characterised by FT-IR, ^13^C NMR, ^1^H NMR, LC-MS and elemental analysis. Their inhibitory effect on AChE, and carbonic anhydrase I and II enzyme activities was investigated. Their antioxidant activity was determined using different bioanalytical assays such as radical scavenging tests with ABTS^•+^, and DPPH^•+^ as well as metal-reducing abilities with CUPRAC, and FRAP assays. All compounds showed satisfactory enzyme inhibitory potency in nanomolar concentrations against AChE and CA isoforms with *K*_I_ values ranging from 10.14 ± 0.03 to 100.58 ± 1.90 nM. Amine group containing derivatives showed high metal reduction activity and about 70% ABTS radical scavenging activity. Due to their antioxidant activity and AChE inhibition, these novel compounds may be considered as leads for investigations in neurodegenerative diseases.

## 1. Introduction

Free radicals are molecules with unpaired electrons resulting from biochemical redox reactions that occur during cell metabolism^1^^,^^2^. The free radicals, which cause oxidation, affect important biomolecules such as lipids, proteins, DNA and carbohydrates, and cause the disruption of their structure^3^^,^^4^. Among the reactive oxygen species (ROS) produced in biological systems, free radicals such as hydroxyl radical (OH^•^), nitric oxide (NO) and peroxyl radical (RCOO^•^) are the most important factors in the induction of oxidative stress^5^. Cells can normally reduce moderate oxidative stress through the antioxidant defence system. However, when oxidative stress reaches high levels, oxidative damage may occur in the cell if adaptation to oxidation products cannot be achieved. The association of oxidative damage of all biomolecules including protein, DNA and lipids with many diseases such as diabetes mellitus, cardiovascular, neurodegenerative and cancer increased the interest in antioxidant studies^6–9^. It is known that enzymatic and non-enzymatic antioxidant systems play an important role in the elimination of free radicals and metabolic products and in the prevention of various diseases and maintenance of normal cellular physiology^10^^,^^11^. Antioxidants continue to attract attention because of their importance in the prevention and treatment of diseases such as coronary heart and neurodegenerative. The antioxidants can be defined as substances that prevent or delay the oxidation of a substance, although it is less common than an oxidisable substance in the same medium. Phenolic compounds, which have an important role in antioxidant activity, remove free radicals and prevent tissue damage due to their chemical structure containing a hydroxyl group (–OH) directly linked to an aromatic hydrocarbon ring^12^^,^^13^. It is known that the increase in the activity of many metabolically important enzymes may cause increased oxidative stress. Therefore, it is of great importance to design and develop new inhibitors for such enzymes. Pharmacological inhibitors of mitochondrial carbonic anhydrase have been reported to be useful in protecting against oxidative stress, which is an important cause in the development of many diseases^14^. Furthermore, the increase in the activity of the neurotransmitter acetylcholine hydrolysing AChE plays a role in the formation of β-amyloid (Aβ) deposited in extracellular toxic plaques in the brains of Alzheimer’s patients^15^^,^^16^.

Many known synthetic antioxidants are most commonly used in food additives and pharmaceutical additives to prevent oxidation. In recent years, many side effects of synthetic antioxidants have raised concerns. In this case, there is a worldwide trend towards the use of safe antioxidants. Therefore, it is important to find new sources for the synthesis of safer and inexpensive antioxidants^17–19^.

Some sulphonamide derivatives were screened for their antioxidant activity, which showed good results^20–23^. Sulphonamides chemically containing sulfamoyl (–SO_2_NH–) group are derivatives of amide. The first sulphonamide drug identified in 1932 was the prontosil and used as antibacterial agent and since then sulphonamides are most widely used in the world among groups of anti-infectives. Sulphonamides are a biologically significant group of compounds due to well absorption orally and excrete in urine, thus sulphonamides have less toxicity, increased reactivity and are cost-effective molecules^24–26^. Today, sulphonamides are widely used as antimicrobial^27^^,^^28^, anti-inflammatory^29^^,^^30^, anticancer^31–36^ and anti-viral agents as well as HIV protease inhibitor^37^, anti-obesity^38^, anti-thyroid^39^, and also act as a potent Carbonic anhydrase *h*CA inhibitors^40^^,^^41^.

There is considerable interest in the chemistry of Schiff base compounds by virtue of having applications in biological, industrial, pharmaceutical and many other fields of science^42^. Sulphonamide Schiff base derivatives could be obtained by condensation of sulphonamide compounds with at least –NH_2_ group and aldehyde and this could led to biologically active compounds^43^. Schiff base derivatives obtained from sulfo drugs drug have drawn attention due to their biological properties^44^.

Here we report the synthesis of compounds having sulphonamide and Schiff base moieties. By keeping in mind, the immense biological significance of sulphonamides and Schiff bases, novel derivatives were synthesised. The inhibitory effect of newly synthesised compounds on *h*CA I, *h*CA II, and AChE enzyme activities were investigated and then antioxidant activity was determined using radical scavenging tests with ABTS^•+^, and DPPH^•+^ and metal-reducing abilities with CUPRAC, and FRAP assays.

## 2. Methods and materials

### 2.1. General

All the chemicals were obtained from commercial suppliers (Merck, Sigma-Aldrich) and were used as received. Diethyl ether, chloroform, tetrahydrofuran (THF), Dichloromethane (DCM), dimethylformamide (DMF), methanol and ethanol were used as solvents. Reagents used were 3-Aminobenzenesulfonamide, 4-Aminobenzenesulfonamide, 3-Bromo-2-hydroxybenzaldehyde and 3-Chloro-2-hydroxybenzaldehyde, acetic acid and formic acid. Potassium ferrocyanide, trichloroacetic acid (TCA), iron III chloride, ammonium acetate, neocuproine, 1,1-Diphenyl-2-picrylhydrazine and 2,2′-Azino-bis(3-ethylbenzothiazoline-6-sulfonic acid was used for antioxidant studies.

### 2.2. Instrumentation and measurements

Elemental analyses were carried out using a LECO CHNS-932 Elemental Analyser. FT-IR spectra were recorded on a Perkin Elmer Spectrum Two FT-IR Spectrometer in the region 400–4000 cm^−1^. A Shimadzu UV-1208 UV-Vis Spectrophotometer was used for the absorption spectra measurements of range 200–1100 nm in DMF at room temperature. NMR spectra were recorded on an Agilent 400 MR (^1^H NMR 400 MHz and ^13^C NMR 100 MHz) in DMSO-*d*_6_, with TMS as an internal standard, at 25 °C. Melting points of compounds were measured in open capillary tubes using an SMP3, Stuart Scientific Melting Point Apparatus. A Shimadzu LC MS 8040 Model Spectrometer was used for recording mass spectra. The course of reactions and product purities were assessed using TLC plates (Merck Silica Gel 60 F_254_).

### 2.3. General procedure for the synthesis of imine derivatives (1–4)

To synthesise imino-derivatives, aromatic aldehyde derivatives (10 mmol) were dissolved in methanol (30 ml) and these solutions were added dropwise to the relevant sulphonamide solutions (10 mmol) dissolved in methanol (30 ml). A catalytic amount of formic acid was added, and the reaction stirred for 3–5 h under reflux. Reactions were monitored through IR spectroscopy and TLC, after completion the solvent was evaporated. The obtained solid was washed with ice-cold ethanol. Then, the obtained products were recrystallized from methanol/ethanol and dried under vacuum to give the corresponding products.

#### 2.3.1. 3-((3-Bromo-2-hydroxybenzylidene)amino)benzenesulfonamide (1)


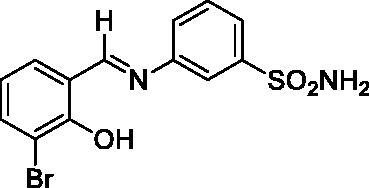


Yield: % 85; Colour: Orange; Melting Point: 206–208°C; Anal. Calcd for C_13_H_11_BrN_2_O_3_S (355.21 g/mol) (%): C, 43.96; H, 3.12; N, 7.89; S, 9.03, Found (%): C, 43.88; H, 3.08; N, 7.99; S, 8.93. FT-IR (U-ATR, υ_max_/cm^−1^): 3296, 3225 (NH_2_), 3130–3360 (O–H⋯N broad), 3085, 3056 (Ar-H), 1611 (–C=N–), 1333 (asymmetric), 1149 (symmetric) (S=O). ^1^H-NMR (DMSO-d_6_, TMS, 400 MHz, δ ppm): 14.05 (1H, s, Ar-OH), 9.05 (1H, s, –CH=N–), 7.44 (2H, s, –SO_2_NH_2_), 7.87 (1H, s, Ar-H), 7.77–7.64 (4H, m, Ar-H), 7.01–6.89 (2H, m, Ar-H). ^13^C-NMR (DMSO-d_6_, TMS, 100 MHz, δ ppm): 165.38 (–C=N–), 157.63 (Ar-C-OH), 147.68 (Ar-C-N), 145.86 (Ar-C–SO_2_NH_2_), 137.23, 133.69, 131.28, 125.71, 124.80, 121.55, 120.33, 118.99, 110.42 (Aromatic Carbons). LC-MS Mass (m/z): Monoisotopic Mass: 353.97; [M + H]^+^: 355.00

#### 2.3.2. 3-((3-Chloro-2-hydroxybenzylidene)amino)benzenesulfonamide (2)


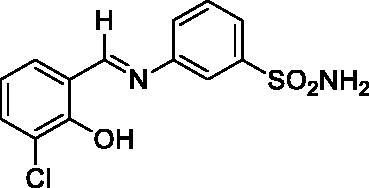


Yield: % 85; Colour: Dark yellow; Melting Point: 205–207°C; Anal. Calcd for C_13_H_11_ClN_2_O_3_S (310.76 g/mol) (%): C, 50.24; H, 3.57; N, 9.01; S, 10.32, Found (%): C, 50.18; H, 3.52; N, 9.12; S, 10.21. FT-IR (U-ATR, υ_max_/cm^−1^): 3359, 3266 (NH_2_), 3115–3410 (O–H⋯N broad), 3085, 3061 (Ar-H), 1617 (–C=N–), 1282 (asymmetric), 1139 (symmetric) (S=O). ^1^H-NMR (DMSO-d_6_, TMS, 400 MHz, δ ppm): 13.89 (1H, s, Ar-OH), 9.07 (1H, s, –CH=N–), 7.44 (2H, s, –SO_2_NH_2_), 7.87 (1H, s, Ar-H), 7.78–7.76 (1H, d, *J =* 8, Ar-H), 7.72–7.64 (3H, m, Ar-H), 7.60–7.58 (1H, d, *J =* 8, Ar-H), 7.02–6.98 (1H, t, *J =* 8, Ar-H).^13^C-NMR (DMSO-d_6_, TMS, 100 MHz, δ ppm): 165.46 (–C=N–), 156.69 (Ar-C-OH), 147.82 (Ar-C-N), 145.84 (Ar-C–SO_2_NH_2_), 134.49, 132.94, 131.31, 130.28, 125.67, 124.91, 121.02, 120.46, 119.43 (Other Aromatic Carbons). LC-MS Mass (m/z): Monoisotopic Mass: 310.02; [M + H]^+^: 311.00

#### 2.3.3. 4-((3-Bromo-2-hydroxybenzylidene)amino)benzenesulfonamide (3)


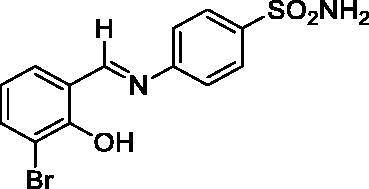


Yield: % 85; Colour: Red; Melting Point: 199–201 °C; Anal. Calcd for C_13_H_11_BrN_2_O_3_S (355.21 g/mol) (%):C, 43.96; H, 3.12; N, 7.89; S, 9.03, Found (%): C, 43.89; H, 3.08; N, 7.96; S, 8.97. FT-IR (U-ATR, υ_max_/cm^−1^): 3303, 3238 (NH_2_), 3140–3420 (O-H⋯N broad), 3064, 3038 (Ar-C-H), 1616 (–C=N–), 1327 (asymmetric), 1158 (symmetric) (S=O). ^1^H-NMR (DMSO-d_6_, TMS, 400 MHz, δ ppm): 14.06 (1H, s, Ar-OH), 9.04 (1H, s, -CH=N–), 7.42 (2H, s, –SO_2_NH_2_), 7.89–7.87 (2H, d, *J =* 8, Ar-H), 7.76–7,74(1H, d, *J =* 8, Ar-H), 7.68–7.66 (1H, d, *J =* 8, Ar-H), 7.63–7.61 (2H, d, *J =* 8, Ar-H), 6.97–6.93 (1H, t, *J =* 8, Ar-H). ^13^C-NMR (DMSO-d_6_, TMS, 100 MHz, δ ppm): 165.88 (–C=N–), 157.78 (Ar-C-OH), 149.96 (Ar-C-N), 142.96 (Ar-C–SO_2_NH_2_), 137.13, 133.28, 127.55, 123.03, 121.84, 120.19, 110.49 (Other Aromatic Carbons). LC-MS Mass (m/z): Monoisotopic Mass: 353.97; [M + H]^+^: 355.00

#### 2.3.4. 4-((3-Chloro-2-hydroxybenzylidene)amino)benzenesulfonamide (4)


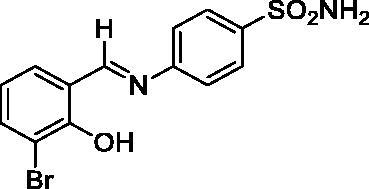


Yield: % 85; Colour: Red; Melting Point: 207–209 °C; Anal. Calcd for C_13_H_11_ClN_2_O_3_S (310.76 g/mol) (%): C, 50.24; H, 3.57; N, 9.01; S, 10.32, Found (%): C, 50.18; H, 3.52; N, 9.10; S, 10.24. FT-IR (U-ATR, υ_max_/cm^−1^): 3311, 3230 (NH_2_), 3140–3400 (O-H⋯N broad), 3064, 3032 (Ar-C-H), 1616 (–C=N–), 1330 (asymmetric), 1162 (symmetric) (S=O). ^1^H-NMR (DMSO-d_6_, TMS, 400 MHz, δ ppm): 13.89 (1H, s, Ar-OH), 9.04 (1H, s, -CH=N–), 7.41 (2H, s, –SO_2_NH_2_), 7.90–7.88 (2H, d, *J =* 8, Ar-H), 7.63–7.58 (4H, m, Ar-H) , 7.02–6.98 (1H, t, *J =* 8, Ar-H). ^13^C-NMR (DMSO-d_6_, TMS, 100 MHz, δ ppm): 165.85 (–C=N–), 156.83 (Ar-C-OH), 150.09 (Ar-C-N), 142.96 (Ar-C–SO_2_NH_2_), 134.52, 131.96, 127.42, 122.99, 121.92, 120.64, 119.40 (Other Aromatic Carbons). LC-MS Mass (m/z): Monoisotopic Mass: 310.02; [M + H]^+^: 311.00

### 2.4. General procedure for the synthesis of amine derivatives (5–8)

To synthesise amino-derivatives (**5–8**), sodium borohydride (NaBH_4_) (70 mmol) was added in small portions to imino-compounds (**1–4**) (10 mmol) dissolved in methanol (60 ml) at 0 °C, over 1 h. The mixture was left under stirring for 24 more hours at room temperature and reductions were monitored through IR spectroscopy and TLC. After the reduction was complete, half of the solvent in the reaction mixture was evaporated and the remaining mixture was poured on ice than the precipitate was filtered and extracted with DCM and chloroform. The solvent was then evaporated and the obtained products were recrystallized from ethanol/methanol (30/70) and dried under vacuum.

#### 2.4.1. 3-((3-Bromo-2-hydroxybenzyl)amino)benzenesulfonamide (5)


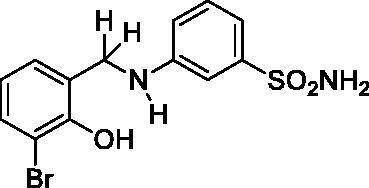


Yield: % 60; Colour: White; Melting Point: 123–124 °C; Anal. Calcd for C_13_H_13_BrN_2_O_3_S (357.22 g/mol) (%): C, 43.71; H, 3.67; N, 7.84; S, 8.98, Found (%): C, 43.63; H, 3.58; N, 7.93; S, 8.91. FT-IR (U-ATR, υ_max_/cm^−1^): 3355, 3335 (NH, NH_2_), 3255 (OH), 3114, 3066 (Ar-H), 2840–2985 (Aliphatic –C–H), 1611 (–C=N–) (disappeared), 1330, 1315 (asymmetric), 1155 (symmetric) (S=O). ^1^H-NMR (DMSO-d_6_, TMS, 400 MHz, δ ppm): 3.00–5.50 (2H, broad, Ar-OH and N-H), 7.32–7.30 (1H, d, *J =* 8, Ar-H), 7.18–7.14 (1H, t, *J =* 8, Ar-H), 7.10–7.08 (1H, d, *J =* 8, Ar-H), 7.18–7.10 (2H, broad, –SO_2_NH_2_), 7.02 (1H, s, Ar-H), 6.93–6.91 (1H, t, *J =* 8, Ar-H), 6.67–6.65 (1H, t, *J =* 8, Ar-H), 6.62–6.59 (1H, t, *J =* 8, Ar-H), 4.29 (2H, s, Ar-CH_2_). ^13^C-NMR (DMSO-d_6_, TMS, 100 MHz, δ ppm): 153.59 (Ar-C-OH), 149.26 (Ar-C-N), 145.13, 131.79, 130.25, 129.23, 128.03, 120.14, 114.52, 113.62, 112.43, 109.74 (Other Aromatic Carbons), 42.81 (Ar-CH_2_-N). LC-MS Mass (m/z): Monoisotopic Mass: 355.98; [M + H]^+^: 357.00

#### 2.4.2. 3-((3-Chloro-2-hydroxybenzyl)amino)benzenesulfonamide (6)


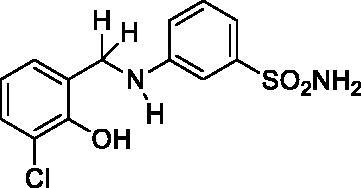


Yield: % 60; Colour: White; Melting Point: 121–122 °C; Anal. Calcd for C_13_H_13_ClN_2_O_3_S (312.77 g/mol) (%): C, 49.92; H, 4.19; N, 8.96; S, 10.25, Found (%): C, 50.01; H, 4.12; N, 9.01; S, 10.16. FT-IR (U-ATR, υ_max_/cm^−1^): 3344, 3324 (NH, NH_2_), 3253 (OH), 3111, 3078 (Ar-C-H), 2860–2995 (Aliphatic –C–H), 1617 (–C=N–) (disappeared), 1325, 1315 (asymmetric), 1153, 1138 (symmetric) (S=O). ^1^H-NMR (DMSO-d_6_, TMS, 400 MHz, δ ppm): 3.00–5.00 (2H, broad, Ar-OH and N-H), 7.22–7.16 (4H, m, Ar-H and –SO_2_NH_2_), 7.11–7.09 (1H, d, *J =* 8, Ar-H), 7.02 (1H, s, Ar-H), 6.96–6.94 (1H, t, *J =* 8, Ar-H), 6.78–6.74 (1H, t, *J =* 8, Ar-H), 6.68–6.66 (1H, t, *J =* 8, Ar-H), 4.27 (2H, s, Ar-CH_2_). ^13^C-NMR (DMSO-d_6_, TMS, 100 MHz, δ ppm): 151.03(Ar-C-OH), 149.20 (Ar-C-N), 145.17, 130.28, 129.00, 127.46, 121.10, 119.83, 114.52, 113.34, 112.31, 109.62 (Other Aromatic Carbons), 42.18 (Ar-CH_2_-N). LC-MS Mass (m/z): Monoisotopic Mass: 312.03; [M + H]^+^: 313.00

#### 2.4.3. 4-((3-Bromo-2-hydroxybenzyl)amino)benzenesulfonamide (7)


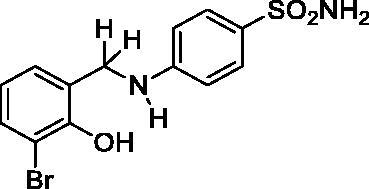


Yield: % 60; Colour: White; Melting Point: 170–172 °C; Anal. Calcd for C_13_H_13_BrN_2_O_3_S (357.22 g/mol) (%):C, 43.71; H, 3.67; N, 7.84; S, 8.98, Found (%): C, 43.62; H, 3.58; N, 7.93; S, 8.93. FT-IR (U-ATR, υ_max_/cm^−1^): 3368, 3348 (NH, NH_2_), 3254 (OH), 3116, 3066 (Ar-C-H), 2840–2985 (Aliphatic –C–H), *1616 (–*C=N–) (disappeared), 1338 (asymmetric), 1135 (symmetric) (S=O). ^1^H-NMR (DMSO-d_6_, TMS, 400 MHz, δ ppm): 3.50–5.00 (2H, broad, Ar-OH and N-H), 6.87 (2H, s, –SO_2_NH_2_), 7.45–7.43 (2H, d, *J =* 8, Ar-H), 7.32–7.30 (1H, d, *J =* 8, Ar-H), 7.07–7.05 (1H, d, *J =* 8, Ar-H), 6.61–6.56 (3H, m, Ar-H), 4.32 (2H, s, Ar-CH_2_). ^13^C-NMR (DMSO-d_6_, TMS, 100 MHz, δ ppm): 153.97 (Ar-C-OH), 151.65 (Ar-C-N), 131.76,.130.48, 129.06, 128.32, 127.15, 119.84, 112.65, 111.12 (Other Aromatic Carbons), 42.47 (Ar-CH_2_-N). LC-MS Mass (m/z): Monoisotopic Mass: 355.98; [M + H]^+^: 357.00

#### 2.4.4. 4-((3-Chloro-2-hydroxybenzyl)amino)benzenesulfonamide (8)


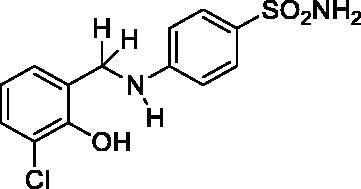


Yield: % 60; Colour: White; Melting Point: 180–182 °C; Anal. Calcd for C_13_H_13_ClN_2_O_3_S (312.77 g/mol) (%): C, 49.92; H, 4.19; N, 8.96; S, 10.25, Found (%):C, 50.01; H, 4.10; N, 9.01; S, 10.16. FT-IR (U-ATR, υ_max_/cm^−1^): 3362 3347 (NH, NH_2_), 3254 (OH), 3125, 3075 (Ar-C-H), 2880–2960 (Aliphatic -C-H), 1616 (–C=N–) (disappeared), 1308 (asymmetric), 1135 (symmetric) (S=O). ^1^H-NMR (DMSO-d_6_, TMS, 400 MHz, δ ppm): 3.50–5.00 (2H, broad, Ar-OH and N-H), 6.88 (2H, s, –SO_2_NH_2_), 7.45–7.43 (2H, d, *J =* 8, Ar-H), 7.16–7.14 (1H, d, *J =* 8, Ar-H), 7.03–7.01 (1H, d, *J =* 8, Ar-H), 6.66–6.62 (1H, t, *J =* 8, Ar-H), 6.58–6.56 (2H, d, *J =* 8, Ar-H), 4.30 (2H, s, Ar-CH_2_). ^13^C-NMR (DMSO-d_6_, TMS, 100 MHz, δ ppm): 153.02 (Ar-C-OH), 151.71 (Ar-C-N), 130.40,129.01, 128.31, 127.18, 121.83, 119.17, 111.70, 111.08 (Other Aromatic Carbons), 42.20 (Ar-CH_2_-N). LC-MS Mass (m/z): Monoisotopic Mass: 312.03; [M + H]^+^: 313.00.

### 2.5. Evaluation of synthesised compounds bioactivity

#### 2.5.1. Metal reducing antioxidant power assay as CUPRAC and FRAP

The power by metal-reducing of novel synthesised sulphonamide derivatives were determined by modified Oyaizu method^45^^,^^46^. Different amounts of derivatives in 1 ml of ethanol were mixed with 0.5 ml of phosphate buffer (0.2 M, pH 6.6) and 0.5 ml of 1% potassium ferricyanide (K_3_Fe(CN)_6_). After the mixtures were incubated at 50 °C for 20 min, trichloroacetic acid (0.5 ml, 10%) was added to each mixture and centrifuged (at 1,008 G for 10 min.). The upper layers of the resulting solutions (0.5 ml) were first mixed with distilled water (0.5 ml) and then FeCl_3_ (0.1 ml, 0.1%) in the given order. absorbances were measured at 700 nm. The high absorbance of the reaction in the mixture shows that the reducing power is increased.

The reduction capacity for cupric ions (Cu^2+^) was determined by Cupric Ions Reducing Assay (CUPRAC) assay as previously described^47^^,^^48^. A volume of 0.25 ml neocuproine (7.5 mM) in ethanol, 0.25 ml NH_4_Ac (1 M) and 0.25 ml CuCl_2_ (0.01 M) was mixed with sample at different amounts and standards.

#### 2.5.2. DPPH^•^ and ABTS^•+^ free radical scavenging activity

The free radical scavenging activity of standard antioxidants and synthesised sulphonamide derivatives was measured by DPPH using the Blois method^49^^,^^50^ 0.1 mM solution of DPPH^•^ in ethanol was prepared and 1 ml of this solution was added to 3 ml of the samples solution in ethanol at different concentrations. These solutions were vortexed thoroughly and kept in the darkness for 30 min. The absorbance values of this final mixture were measured at 517 nm. The reduced absorbance of the reaction mixture indicates higher free radical scavenging activity.

ABTS^•+^ scavenging activity assay was performed according to Re method^51^^,^^52^. The process of ABTS^•+^ (2.0 mM) in water with potassium persulfate (K_2_S_2_O_8_) (2.45 mM) at room temperature in dark for 4 h. gave the ABTS cation radical. Dilution of ABTS^•+^ was applied with sodium phosphate buffer (Na_3_PO_4_) (0.1 mol/l, pH 7.4) to measure the absorbance at 734 nm. The reactions of ABTS^•+^ solution (1.0 ml) with samples solution in ethanol at different concentrations were performed. The inhibition was calculated at 734 nm for each concentration^53^. The DPPH^•^ and ABTS^•+^ free radical scavenging ability was calculated as the following equation: Radical scavenging activity (%) = [(Ac – As)/Ac] × 100. Where, Ac is the absorbance value of DPPH^•^ and ABTS^•+^ before sample addition, while as is the absorbance value after sample addition.

#### 2.5.3. The activity of purified CA isoenzymes

According to our previous studies, the purity and presence of human (h) carbonic anhydrase isoforms purified using Sepharose-4B-L-tyrosine-sulfanilamide affinity chromatography^54^ was tested by SDS-PAGE technique^55^^,^^56^ with Laemmli’s (1970) procedure^57–59^. In the inhibition studies, according to our previous studies, the esterase activities of *h*CA isoforms were determined by the *p*-nitrophenylacetate that used as a substrate converted by both isoforms to the p-nitrophenolate ion in this technique^60–62^.

#### 2.5.4. The activity of anticholinesterase

AChE activity isolated from electric eel (purchased) was performed by a modified version of the Ellman method^63^. The measurement of AChE activity was performed using Acetylthiocholine (ATChI) iodide as the substrates and 5,5-Dithiobis(2-nitrobenzoic) acid (DTNB)^64^. The ATChI iodide as the substrates was monitored spectrophotometrically at 412 nm as in our previous assays^65^^,^^66^.

#### 2.5.5. *In vitro* inhibition study

The inhibition effects of synthesised derivatives were determined with least five different inhibitor concentrations on *h*CA isoenzymes and AChE. *IC*_50_ of synthesised compound was calculated from Activity (%)-[synthesised derivatives] graphs for each compound. The inhibition types and *K*_I_ values were found by Lineweaver and Burk’s (1934) curves^67^^,^^68^.

### 2.6. Computational study

Qikprop software^69^, included in the Schrodinger Suite 2019–4, was used to foresee pharmaceutically relevant various ADMET, and drug-likeness parameters of the synthesised sulphonamide imine, and amine compounds (**1**–**8**), such as number of violations of Jorgensen’s rule of three^70^, and number of violations of Lipinski’s rule of five^71^ that are significant in the novel drug discovery and development process.

The X-ray crystallographic structure of *h*CA I, II, and AChE co-crystallized with 3UF, V50, and E20 (PDB ID: 4WUP 1.75 Å^72^, 4HT0 1.60 Å^73^ and 4EY7 2.35 Å^74^, respectively) were accessed from the protein data bank (rcsb.org)^75^. The proteins were prepared for the docking work utilising the Protein Preparation Wizard ^76^ in Maestro with default options (Schrodinger Suite 2019–4). The 3 D structures of the ligands (1–8) were drawn by using the ChemDraw software and were prepared using the LigPrep platform^77^, considering their ionisation state at pH 7.0 ± 0.5^78^ with Epic. The energy minimises of the receptors and ligands (**1**–**8**) were conducted using OPLS3e force field protocol^79^. The docking grid was centred on the centre of mass of the co-crystallized ligands (3UF, V50 and E20) and was built using the Receptor Grid Generation tool^80^. The molecular docking experiment was accomplished for all the synthesised agents against target receptors by using the Glide extra precision (XP) algorithm^81^.

### 2.7. Statistical study

Analysis of the data, and drawing of graphs were realised using GraphPad Prism version 6 for Mac, GraphPad Software, La Jolla, CA. Also, the determination of *K*_I_ constants was conducted using SigmaPlot version 12, from Systat Software, San Jose, CA. The results were exhibited as mean ± standard deviation (95% confidence intervals). Differences between data sets were considered as statistically significant when the *p* values was less than 0.05.

## 3. Results and discussion

### 3.1. Synthesis of sulphonamide imine (1–4) and amine (5–8) compounds

**Scheme 1.**
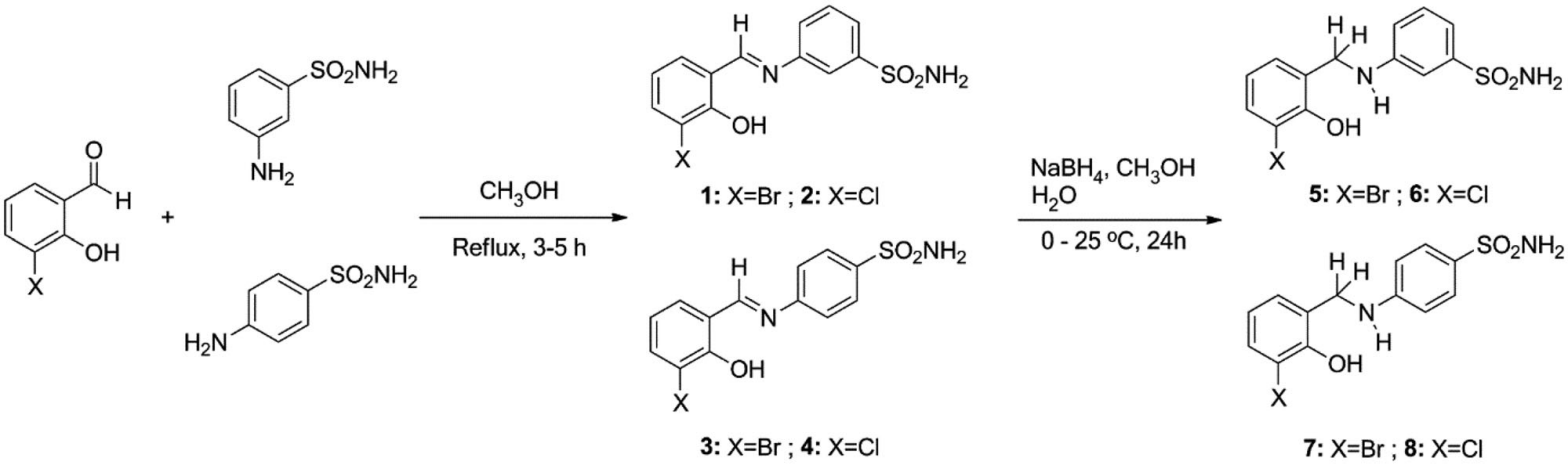
General synthetic procedure for target analogues (**1**–**8**).

In this study, the sulphonamides containing imine group (**1–4**) were synthesised and then, the secondary amine sulphonamides (**5**–**8**) were obtained through reduction of the imine derivatives (**1**–**4**) according to literature methods^82^. 3-Aminobenzenesulfonamide and 4-aminobenzenesulfonamide were condensed with 3-bromo-2-hydroxybenzaldehyde and 3-chloro-2-hydroxybenzaldehyde in the presence of catalytic amounts of formic acid. In separate reactions, imine compounds synthesised as above were then reduced with NaBH_4_ to obtain the novel secondary amine sulphonamides. The synthesis of the imine (**1**–**4**) and amine (**5**–**8**) sulphonamide compounds are illustrated in Scheme 1. The synthesised imine (**1**–**4**) and amine (**5**–**8**) derivatives were obtained as solid products, stable at room temperature.

FT-IR, ^1^H NMR, ^13^C NMR, LC-MS-MS and elemental analysis were performed to know the exact nature of functional groups, the arrangement of protons and carbons, the molecular mass and fragmentation pattern and the percentage of the constituting elements respectively. In addition, their purity was investigated with these methods and it was determined that they did not contain any residue. The sulphonamide derivatives, imine (**1**–**4**) and amine (**5**–**8**) compounds, were in good agreement with calculated values. Data given in the experimental section are in complete agreement with those of previous studies for other such sulphonamide derivatives^42^^,^^82–85^.

#### 3.1.1. Fourier transform infra-red spectroscopy (FT-IR) measurements

FT-IR spectra of starting materials, imine and amine compounds were obtained via U-ATR at range 400–4000 cm^−1^ and were used to give particular information on spectroscopic characterisation of the compounds. All sulphonamides showed characteristic vibrations at ranges of 3324–3368 cm^−1^ for -NH_2_, 3032–3116 cm^−1^ for aromatic C-H and 1135–1338 cm^−1^ for S=O. Due to the strong intramolecular interactions between –OH and –C=N– groups in the compound, O–H stretching peak could not be observed in the region expected (∼3300 cm^−1^). This band observed at 3100–3400 cm^−1^. As evidence of the reduction of imines (**1**–**4**) characteristic vibrations at ranges of 1611–1617 cm^−1^ for –C=N– weren’t observed in the reduced compounds (**5**–**8**). Also –N–H vibrations at ranges of 3324–3368 cm^−1^ and aliphatic –CH vibrations at ranges of 2840–2985 cm^−1^ were observed in the reduced compounds (**5**–**8**) whereas were not detected at imine compounds (**1**–**4**).

#### 3.1.2. ^1^H and ^13^C NMR spectroscopic analyses

The NMR spectra of all compounds were recorded in DMSO-d_6_ with TMS as an internal standard and data given in the experimental section.

The ^1^H NMR spectra of all compounds exhibited singlets at δ 6.87–7.44 ppm for sulphonamide protons (–SO_2_NH_2_) and singlets for aromatic ring protons at δ 7.02–7.87 ppm. Imine compounds (**1**–**4**) gave singlets at δ 13.89–14.06 ppm which being attributed aromatic –OH protons. Singlets for aromatic ring protons were observed at δ 7.02–7.87 ppm besides singlets for imine proton (–CH=N) were observed at δ 9.04–9.07 which were unseen in the reduced compounds (**5**–**8**). Also, as another proof of reduction of the imine compounds (**1**–**4**), singlets were seen at δ 4.29–4.32 ppm, which were attributed to Ar-CH_2_ group in the reduced compounds (**5**–**8**). A broad signal was observed at amine compounds (**5**–**8**) in the region δ 4.29–4.32 ppm arising from –NH protons could admit another evidence for reduction of imine compounds (**1**–**4**). Doublet peaks were observed for aromatic proton (Ar-H) groups, both neighbouring single protons. Multiplet peaks were often observed and these represented the aromatic protons (Ar-H) of benzenesulfonamide ring and aromatic aldehyde ring for all derivatives.

The ^13^C NMR spectra of all compounds gave aromatic carbons in the region δ 109.62–145.17 ppm deriving from benzenesulfonamide ring and aromatic aldehyde ring. The imine carbon (–C=N–) at imine compounds (**1**–**4**) was observed in the region δ 165.38–165.88 ppm which unseen in the reduced compounds (**5**–**8**). A methylene carbon (Ar-CH_2_-N) at amine compounds (**5**–**8**) observed at δ 42.20–42.81 ppm could be a sign for reduction of imine compounds (**1**–**4**).

#### 3.1.3. Mass spectra (LC-MS-MS)

LC-MS was used to obtain the molecular masses of newly synthesised imine compounds (**1**–**4**) and amine compounds (**5**–**8**) and spectra were used as evidence for the formation of the proposed structures. Synthesised compounds support the suggested structures and molecular ions [M]^+^ of the derivatives were detected as single sharp peaks and observed as [M + H]^+^ ions. All spectra were in agreement with the molecular structures of the derivatives and the values of molecular weights supplied by mass spectrometer given in the experimental section.

### 3.2. Biological evaluation

CA isoenzymes (*h*CA I and *h*CA II isoenzymes), which play an important role in many biochemical and physiological processes in living organisms and have a higher catalytic rate in the cytosol of erythrocytes, play a key role in biosynthetic reactions such as ureagenesis and lipogenesis^86^^,^^87^. In recent years, it has been known that diuretics, antiglaucoma, antiobesity, antitumor and anticonvulsant agents have been used as *h*CA inhibitors in the treatment of many disease symptoms^88^.

Specific inhibitors of *h*CA I, and II isoenzymes have been used for the treatment of several diseases in clinic such as glaucoma, duodenal and gastric ulcers, epilepsy, congestive heart failure, mountain sickness, and as diuretic agents. However, clinically used CAIs show side effects due to lack of isoenzyme selectivity of the compounds. In this study, it was determined the effect of novel sulphonamides for the inhibition of two physiologically relevant CAs, the cytosolic isoforms *h*CA I and II as well as AChE. As the reference inhibitor, acetazolamide (AZA) was utilised for *h*CA I, and II, and tacrine (TAC) was used for AChE.

*h*CA I was strongly inhibited by Schiff bases sulphonamides **1**–**8**. The secondary amines group (compounds **5**–**8**) showed a potent inhibitory effect, according to the imine group (compounds **1**–**4**). These compounds demonstrated *K*_I_ values in the low nanomolar range (*K*_I_ values ranging from 32.1 ± 0.4 to 100.6 ± 1.9 nM). The order of the *K*_I_ values of the sulphonamides was **8** (*K*_I_: 32.1 ± 0.4 nM) > **4** (*K*_I_: 40.1 ± 0.6 nM) > **5** (*K*_I_: 52.1 ± 0.8 nM) > **1** (*K*_I_: 59.7 ± 0.8 nM) > **7** (*K*_I_: 62.3 ± 1.3 nM) > **2** (*K*_I_: 66.6 ± 1.0 nM) > **3** (*K*_I_: 80.5 ± 1.5 nM) > **6** (*K*_I_: 100.6 ± 1.9 nM) (Table 1). Compound **8** showed the best inhibition profile against *h*CA I compared to that of a standard drug, AAZ (*K*_I_: 436.2 ± 12.2 nM, approx. 13.5-fold) (Table 2). On the other hand, the least effective compound was **6** with *K*_I_ of 100.6 ± 1.9 nM.

**Table 1.** *K*_I_ values of *h*CA I, II and AChE with derivatives **1–8**, AAZ and TAC as standard inhibitors.

Compounds	*h*CA I		*h*CA II		AChE	
	*K*_I_ (nM)	*R*^2^	*K*_I_ (nM)	*R*^2^	*K*_I_ (nM)	*R*^2^
**1**	59.7 ± 0.1	0.9993	16.4 ± 0.7	0.9995	24.3 ± 1.0	0.9995
**2**	66.5 ± 0.9	0.9991	13.8 ± 0.1	0.9999	21.00 ± 0.9	0.9994
**3**	80.5 ± 1.5	0.9991	26.3 ± 0.09	0.9999	34.9 ± 1.4	0.9996
**4**	40.1 ± 0.6	0.9988	79.3 ± 0.2	0.9999	27.9 ± 1.3	0.9994
**5**	52.1 ± 0.7	0.9992	19.8 ± 1.2	0.9993	61.2 ± 4.0	0.9993
**6**	100.6 ± 1.9	0.9984	29.1 ± 0.3	0.9999	25.7 ± 1.0	0.9995
**7**	62.32 ± 1.3	0.9981	10.1 ± 0.03	0.9999	77.0 ± 9.3	0.9984
**8**	32.1 ± 0.4	0.9991	61.9 ± 0.2	0.9999	31.4 ± 1.2	0.9996
AAZ^a^	436.2 ± 12.2	0.9982	93.5 ± 1.2	0.9996	–	–
TAC^b^	–	–	–	–	109.75 ± 2.39	0.9999

^a^Acetazolamide. ^b^Tacrine.

**Table 2.** Selectivity index values for *K*_I_ values of the compounds **1**–**8**.

Compounds	*K*_I_ (*h*CA II/*h*CA I)	*K*_I_ (AAZ^a^/*h*CA I)	*K*_I_ (AAZ^a^/*h*CA II)	*K*_I_ (TAC^b^/AChE)
**1**	0.27	7.31	5.72	4.52
**2**	0.21	6.55	6.77	5.23
**3**	0.33	5.42	3.55	3.14
**4**	1.98	10.89	1.18	3.93
**5**	0.38	8.37	4.72	1.79
**6**	0.29	4.34	3.22	4.28
**7**	0.16	7.00	9.22	1.42
**8**	1.93	13.57	1.51	3.50

^a^Acetazolamide.

^b^Tacrine.

The physiologically most abundant cytosolic isozyme *h*CA II was inhibited by many of the compounds in the range of *K*_I_ value of 10.1 ± 0.1–79.3 ± 0.2 nM (Table 1). The order of the *K*_I_ values of the inhibitory strength of the sulphonamides was as **7** (*K*_I_: 10.1 ± 0.1 nM) > **2** (*K*_I_: 13.8 ± 0.1 nM) > **1** (*K*_I_: 16.4 ± 0.8 nM) > **5** (*K*_I_: 19.8 ± 1.2 nM) > **3** (*K*_I_: 26.3 ± 0.1 nM) > **6** (*K*_I_: 29.1 ± 0.3 nM) > **8** (*K*_I_: 62.0 ± 0.2 nM) > **4** (*K*_I_: 79.3 ± 0.2 nM). *h*CA II was more weakly inhibited by Schiff bases sulphonamides **1**–**8** as compared to *h*CA I. Although, the secondary amines **5**–**8** were highly effective *h*CA II inhibitors with *K*_I_s in the range of 10.1 ± 0.1–62.0 ± 0.2 nM in comparison with AAZ, a clinical drug (*K*_I_: 93.5 ± 1.2 nM).

The disorder in the cholinergic system is caused by increased activity of AChE catalysing hydrolysis of neurotransmitter acetylcholine. The increase in activity increases the formation of amyloid protein and hydrolysis of acetylcholine, causing neurodegenerative diseases such as Alzheimer’s. Inhibition of AChE has been used in the treatment of some symptoms that may occur with excessive hydrolysis of ACh and increased amyloid proteins^89^. Many synthetic and natural substances in metabolism can affect the metabolic pathway by modifying enzyme activities at low concentrations. Inhibition of AChE by novel synthesis compounds is currently important to improve the effective treatment of AD^90^.

Since sulphonamides were reported with their significant inhibitory potency on AChE enzyme, which is a well-known therapeutic target of Alzheimer’s disease, novel sulphonamides in this study were screened on AChE enzyme. The *K*_I_ values of the compounds (**1**–**8**) were given in Table 1. All of the compounds showed the satisfactory inhibition profile in nanomolar concentrations against AChE which demonstrated *K*_I_ values ranging from 21.0 ± 0.9 to 77.0 ± 9.3 nM when compared to TAC (*K*_I_: 109.8 ± 2.4 nM). The order of the *K*_I_ constants of the inhibitory strength of the sulphonamides was as **2** (*K*_I_: 21.0 ± 0.9 nM) > **1** (*K*_I_: 24.3 ± 1.0 nM) > **6** (*K*_I_: 25.7 ± 1.0 nM) > **4** (*K*_I_: 27.9 ± 1.3 nM) > **8** (*K*_I_: 31.4 ± 1.2 nM) > **3** (*K*_I_: 34.9 ± 1.4 nM) > **5** (*K*_I_: 61.2 ± 4.0 nM) > **7** (*K*_I_: 77.0 ± 9.3 nM). In contrast to *h*CA I and *h*CA II, the secondary amine groups showed less inhibitory effect on AChE enzyme activity according to our results. While compound **2** had the best inhibition profile against AChE, the lowest effective compound was **7**.

The free radical scavenging, metal chelating, metal-reducing capacity of a phenolic acid depends on the hydrogen atom or electron donation in the structure of the compound or the number and position (–OH) of hydroxyl groups^91–93^. Therefore, the structure and functional groups of the compounds are important for their bioactive properties.

In this study, the reducing power of synthesised compounds was investigated by FRAP and CUPRAC assays. An important parameter in the evaluation of antioxidant activity is the reduction of Cu^+2^/Fe^3+^ (ferricyanide) complex to Cu^+1^/Fe^2+^ form. As seen in Table 3, Fe^3+^ reducing ability of standard and synthesised compounds **1–8** decreased in the following order: trolox > BHA > BHT > **6 **>** 7** > **8 **>** 5** > **4 **>** 2** > **1 **>** 3**. According to results obtained from CUPRAC assay, which is based on reduction of Cu^2+^ to Cu^+1^ by synthesised compounds (Table 3). The cupric ion (Cu^2+^) reducing power of standard and synthesised compounds **1**–**8** decreased in the following order: BHA > BHT > trolox > **7 **>** 5** > **6 **>** 8** > **3 **>** 2** > **4 **>** 1**. The compounds **5**–**8** have a higher metal reduction capacity, while the compounds **1**–**4** have a lower metal reduction capacity. Among the synthesised compounds **(1–8)**, the compounds that are reduced and contain secondary amine groups **(5–8)** have a higher reduction capacity compared to others **(1–4)**. This may be because the reduced compounds **(5–8)** contain extra N-H bonds compared to normal Schiff bases **(1–4)**.

**Table 3.** The radical scavenging and metal reduction activity of synthesised compounds (**1**–**8**).

Compounds	DPPH^a^ [0.05 mg/mL]	ABTS^a^ [0.2 mg/mL]	Fe^3+^ reducing ability^b^ [0.2 mg/mL]	Cu^2+^ reducing ability^b^ [0.2 mg/mL]
**1**	18.7 ± 1.5	18.4 ± 2.0	0.15 ± 0.03	0.22 ± 0.02
**2**	8.2 ± 0.4	15.1 ± 1.4	0.27 ± 0.02	0.26 ± 0.04
**3**	5.9 ± 0.4	7.3 ± 0.6	0.11 ± 0.01	0.28 ± 0.04
**4**	12.7 ± 0.9	14.7 ± 0.9	0.33 ± 0.02	0.24 ± 0.05
**5**	1.9 ± 0.09	76.8 ± 8.1	0.33 ± 0.03	0.99 ± 0.07
**6**	9.2 ± 0.6	77.7 ± 7.9	0.47 ± 0.06	0.93 ± 0.07
**7**	4.2 ± 0.4	74.5 ± 6.4	0.39 ± 0.04	1.15 ± 0.08
**8**	3.2 ± 0.6	78.4 ± 7.7	0.33 ± 0.07	0.80 ± 0.05
BHT^c^	47.0 ± 5.2	98.9 ± 9.1	0.67 ± 0.01	2.00 ± 0.11
BHA^c^	21.7 ± 1.9	79.5 ± 6.1	1.23 ± 0.20	2.28 ± 0.13
Trolox^c^	57.8 ± 6.8	94.4 ± 9.0	1.27 ± 0.12	1.81 ± 0.05

Data are mean ± standard deviation (*n* = 3). BHA: butylated hydroxyanisole; BHT: butylated hydroxytoluene.

^a^The percent (%) of ABTS and DPPH radical scavenging activity.

^b^The values were expressed as absorbance. High absorbance indicates high metal ions (Fe^3+^ and Cu^2+^) reducing ability.

^c^Standard antioxidant.

DPPH^•^, ABTS^•+^, DMPD^•+^ and O_2_^•^ scavenging assays are often used to determine the radical removal activities of synthesised or isolated pure compounds^94^. In this study, the radical scavenging ability of the synthesised compounds **1**–**8** was evaluated by ABTS^•+^ and DPPH^•^ scavenging assays. The synthesised compounds exhibited radical scavenging activity in range from 7.3 to 78.4% for ABTS at concentration of 200 μg/mL, and in range from 1.9 to 18.7% for DPPH at concentration of 50 μg/mL. As shown in the Table 3, compound **1** to DPPH assay and compound **5–8** for ABTS assay have the ability to remove radicals close to some standards.

In this assay, the synthesised compounds (**5**, **6**, **7**, and **8**), which are primary sulphonamides, showed ABTS radical scavenging activity because they contained both hydroxyl group and nitrogen bound hydrogen. These results clearly demonstrated significant free radical scavenging activity of the newly synthesised sulphonamide compounds **5**–**8**, which have more electron donor properties than others.

### 3.3. Computational study

The ADMET properties, and some pharmacokinetic parameters of the new synthesised sulphonamide imine, and amine compounds (**1**–**8**) were estimated by using the QikProp module. The overall estimated values are summarised in Table 4. As a result, the ADMET study exhibited that these active derivatives **(1–8)** possess the drug-likeness criteria complied by both Jorgensen’s rule of three and Lipinski’s rule of five.

**Table 4.** ADMET–related parameters of the derivatives (**1**–**8**).

Principal descriptors	1	2	3	4	5	6	7	8	Standard range
QPlogPw	13.47	13.46	13.47	13.46	14.84	14.83	14.83	14.82	4 to 45
QPlogPo/w	1.50	1.43	1.50	1.43	1.26	1.16	1.36	1.24	−2.0 to 6.5
QPlogS	−3.45	−3.35	−3.46	−3.36	−3.33	−3.17	−3.42	−3.26	−6.5 to 1.5
QPlogKp	−3.38	−3.39	−3.39	−3.40	−3.43	−3.44	−3.42	−3.43	−8.0 to −1.0
QPlogBB	−1.42	−1.43	−1.42	−1.44	−1.41	−1.43	−1.40	−1.42	−3.0 to 1.2
QPlogKhsa	−0.36	−0.38	−0.36	−0.38	−0.39	−0.40	−0.39	−0.40	−1.5 to 1.5
QPlogHERG	−5.63	−5.60	−5.64	−5.61	−5.56	−5.53	−5.55	−5.52	<−5
HOA	75.69	75.13	75.66	75.09	74.33	73.60	75.03	74.22	<25 poor, great >500
PSA	95.15	95.19	95.20	95.23	98.30	98.31	97.98	98.06	7 to 200
Rule of Five	0	0	0	0	0	0	0	0	max. 4
Rule of Three	0	0	0	0	0	0	0	0	max. 3

Various computational pharmacodynamic and pharmacokinetic parameters of synthesised compounds in this research were predicted such as water/gas partition coefficient (QPlogPw), octanol/water partition coefficient (QPlogPo/w), aqueous solubility (QPlogS), skin permeability (QPlogKp), brain/blood partition coefficient (QPlogBB), prediction of binding to human serum albumin (QPlogKhsa), IC_50_ value for blockage of HERG K^+^ channels (QPlogHERG), human oral absorption (HOA), van der Waals surface area of polar nitrogen and oxygen atoms (PSA), number of violations of Lipinski’s rule of five and number of violations of Jorgensen’s rule of three.

To understand the trends investigated for the monitored relative selectivity of synthesised novel sulphonamide imine, and amine agents (**1**–**8**), the molecular docking study was achieved for the most active selected among the compounds. The X-ray crystal structures of 4WUP and 4EY7 were available in the form of a homodimer chain, therefore, their chain A was chosen for *in silico* works. The 3UF (C_8_H_11_NO_3_S_2_, 4-[(2-hydroxyethyl)sulfanyl]benzenesulfonamide), V50 (C_12_H_9_F_4_N_3_O_2_S_2_, 4-[(4,6-dimethylpyrimidin-2-yl)thio] − 2,3,5,6-tetrafluorobenzenesulfonamide), and E20 (C_24_H_29_NO_3_, 1-benzyl-4-[(5,6-dimethoxy-1-indanon-2-yl)methyl]piperidine) were the co-crystallized ligand with 4WUP (for *h*CA I), 4HT0 (for *h*CA II), and 4EY7 (for AChE), respectively. The *in silico* molecular docking procedure for these receptors was validated by extracting the bound agents (3UF, V50 and E20) from the proteins and again redocking it on similar regions. The docking poses were superimposed, and RMSD values were calculated to be 1.09, 1.28 and 0.08 Å, respectively.

**Figure 1.**
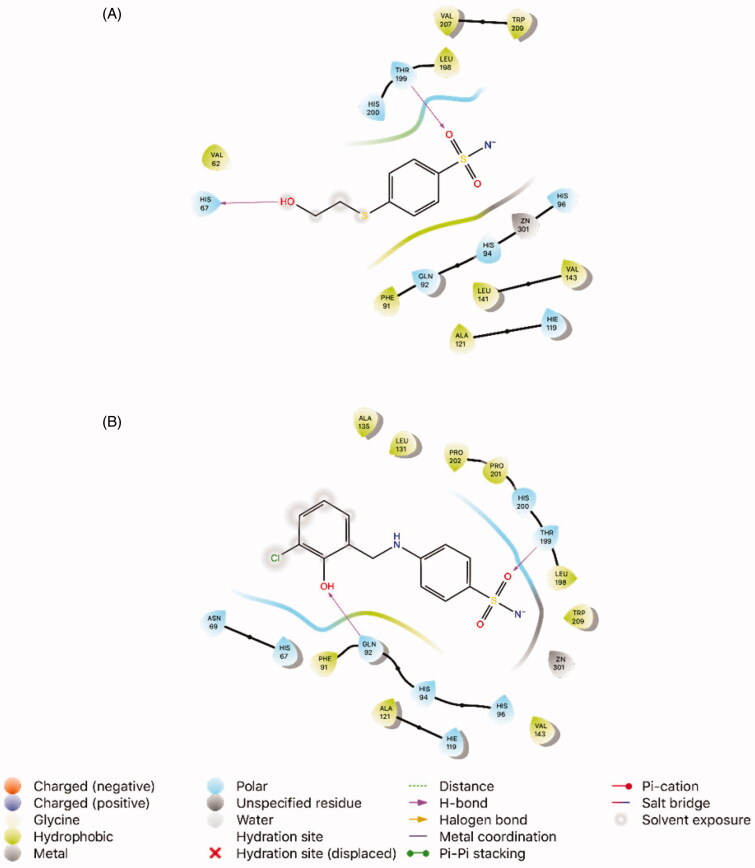
Interaction of the ligands with the key amino acids within the active site of *h*CA I (PDB ID: 4WUP). (A) Docking pose of the native ligand 3UF (4-[(2-hydroxyethyl)sulfanyl]benzenesulfonamide, PubChem CID: 4269754). (B) Docking pose of compound **8** (4-((3-chloro-2-hydroxybenzyl)amino)benzenesulfonamide).

According to the literature, the native ligand (3UF) displays two major interactions, like H-bond interaction with His67, and Thr199 and the docking score was −5.01 kcal/mol, in the catalytic domain of 4WUP For the most active compound (**8**, *K*_I_ 32.14 ± 0.39 nM) the docking score (−6.90 kcal/mol) was found low to the co-crystallized ligand. Compound **8** exhibited H-bond interactions with Gln92, and Thr199 (Figure 1).

**Figure 2.**
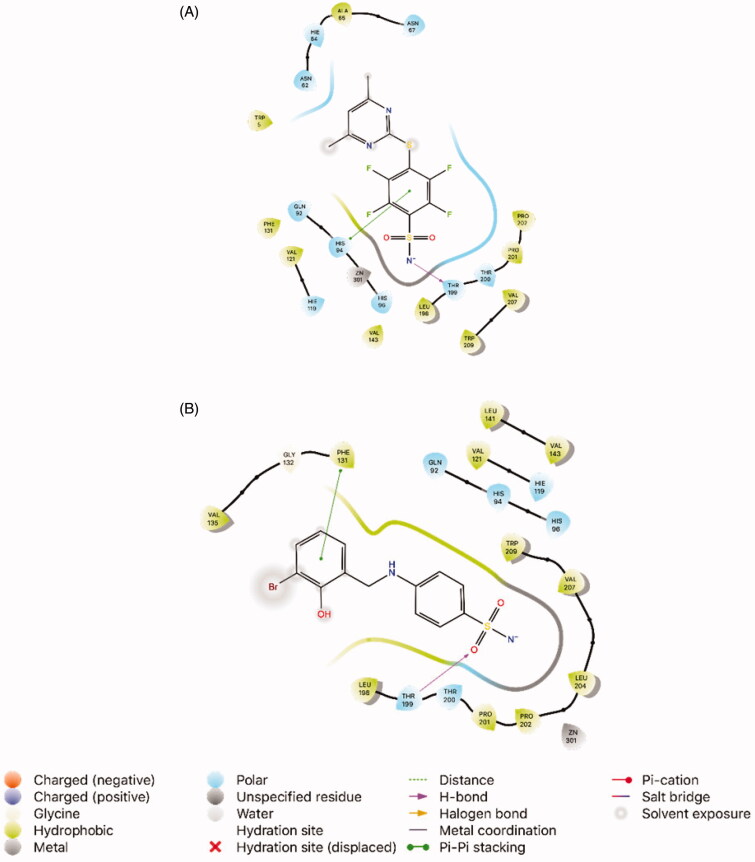
Interaction of the ligands with the key amino acids within the active site of *h*CA II (PDB ID: 4HT0). (A) Docking pose of the native ligand V50 (4-[(4,6-dimethylpyrimidin-2-yl)thio]-2,3,5,6-tetrafluorobenzenesulfonamide, PubChem CID: 71299336). (B) Docking pose of compound **7** (4-((3-bromo-2-hydroxybenzyl)amino)benzenesulfonamide).

The 4HT0 complexed with V50 shows an H-bond with Thr199. Moreover, V50 exhibits pi-pi stacking with His94. The docking score was −4.85 kcal/mol, although it was expected to be in the range of at least −5.00 to −6.00 kcal/mol. As shown in Figure 2, compound **7** (*K*_I_ 10.14 ± 0.03 nM, docking score −6.31 kcal/mol) formed an H-bond with the active site residue (Thr199), and pi-pi stacking with Phe131. Apart from this, this agent showed hydrophobic interactions with Val121, Phe131, Val135, Leu141, Val143, Leu198, Pro201, Pro202, Leu204, Val207 and Trp209.

**Figure 3.**
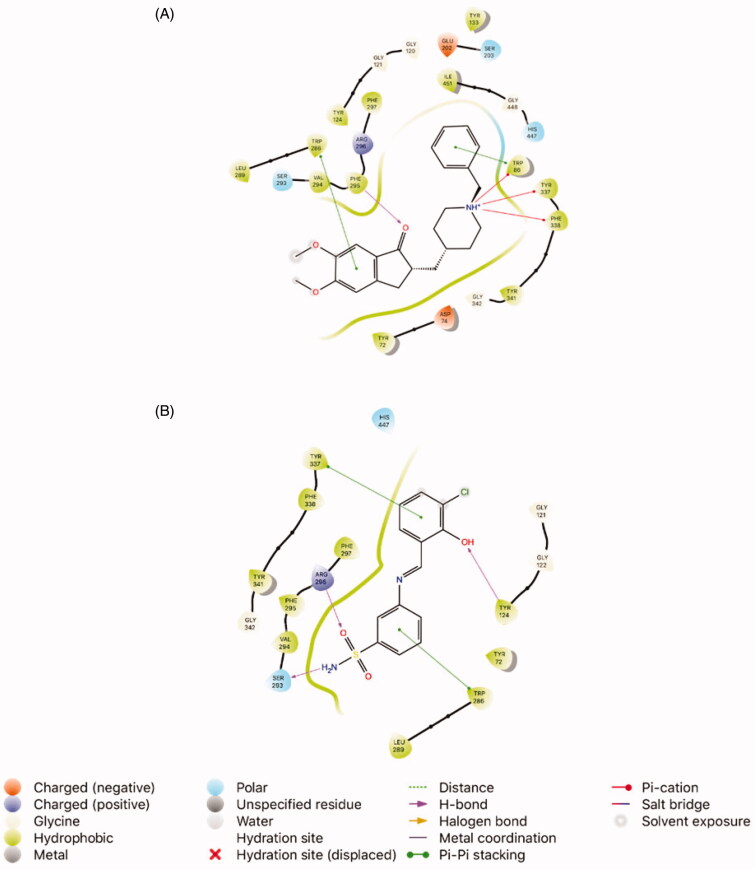
Interaction of the ligands with the key amino acids within the active site of AChE (PDB ID: 4EY7). (A) Docking pose of the native ligand E20 (1-benzyl-4-[(5,6-dimethoxy-1-indanon-2-yl)methyl]piperidine, PubChem CID: 1150567). (B) Docking pose of compound **2** (3-((3-chloro-2-hydroxybenzylidene)amino)benzenesulfonamide).

E20, which has previously considered to be the native ligand, and compound **2** (*K*_I_ 20.98 ± 0.89 nM), which is the most active analogue of the synthesised agents, were analysed in terms of interactions with AChE. The docking positions into 4EY7 determined for the two inhibitors were like to that estimated for their other compounds and both E20 and agent **2** displayed the same interactions with Trp286. Moreover, the docking scores were −16.06 kcal/mol, and −9.26 kcal/mol, respectively. Within the 4EY7 active site, the hydroxy group on the aryl ring, and the amino moiety of the benzenesulfonamide group of compound **2** showed potential two H-bonds with Tyr124 and Ser293, respectively. Also, hydrophobic interaction was monitored between compound **2** and Tyr72, Tyr124, Trp286, Leu289, Val294, Phe295, Phe297, Tyr337, Phe338 and Tyr341. Apart from these, agent **2** also exhibited pi–pi stacking with Trp337 (Figure 3).

## 4. Conclusions

In this study, a series of eight sulphonamide Schiff bases (**1–4**) and their reduced counterparts (**5–8**) were synthesised by the condensation of two well-known sulphonamide derivatives (3-aminobenzenesulfonamide and 4-aminobenzenesulfonamide) with substituted aromatic aldehydes. The obtained novel compounds (**1–8**) were investigated as inhibitors of the cytosolic CA isozymes *h*CA I and *h*CA II, and cholinesterase (AChE) enzymes. The antioxidant activity of the compounds was also performed using different bioanalytical assays such as radical scavenging tests with ABTS^•+^, and DPPH^•+^ and metal-reducing abilities with CUPRAC, and FRAP assays. In general, all compounds showed great inhibition potency against *h*CA I with K_I_ values ranging from 16.05 ± 0.47 to 29.66 ± 0.50 nM. Among the series, one of the most potent inhibition results was observed against hCA II isozyme with compound **7** (K_I_: 10.14 ± 0.03 nM). Another interesting finding from the current study is that all the synthesised compounds showed a better inhibition profile than Tacrine (K_I_: 109.75 ± 2.39) against AChE with K_I_s in between 20.98 ± 0.89 to 77.02 ± 9.34 nM. Also, the reduced derivatives (**5–8**) displayed high metal reduction activity and about 70% ABTS radical scavenging activity. As a result, since AChE inhibition is an important task in the treatment of Alzheimer’s disease, these compounds may have an interesting future for the development of new drugs to neurodegenerative disorders.
